# Does NICE apply the rule of rescue in its approach to highly specialised technologies?

**DOI:** 10.1136/medethics-2020-106759

**Published:** 2021-03-08

**Authors:** Victoria Charlton

**Affiliations:** Global Health and Social Medicine, School of Social Science and Public Policy, King's College London, London, UK

**Keywords:** ethics, allocation of health care resources, distributive justice, public policy, health economics

## Abstract

The National Institute for Health and Care Excellence (NICE), the UK’s main healthcare priority-setting body, recently reaffirmed a longstanding claim that in recommending technologies to the National Health Service it cannot apply the ‘rule of rescue’. This paper explores this claim by identifying key characteristics of the rule and establishing to what extent these are also features of NICE’s approach to evaluating ultra-orphan drugs through its highly specialised technologies (HST) programme. It argues that although NICE in all likelihood does not act because of the rule in prioritising these drugs, its actions in relation to HSTs are nevertheless in accordance with the rule and are not explained by the full articulation of any alternative set of rationales. That is, though NICE implies that its approach to HSTs is not motivated by the rule of rescue, it is not explicit about what else might justify this approach given NICE’s general concern with overall population need and value for money. As such, given NICE’s reliance on notions of procedural justice and its commitment to making the reasons for its priority-setting decisions public, the paper concludes that NICE’s claim to reject the rule is unhelpful and that NICE does not currently meet its own definition of a fair and transparent decision-maker.

## Introduction

The National Institute for Health and Care Excellence (NICE) is the UK’s main healthcare priority-setting body, responsible for making recommendations about what technologies users of the National Health Service (NHS) in England should have access to. NICE’s recommendations are made, prima facie, on the basis of a technology’s cost-effectiveness: the number of additional quality-adjusted life-years (QALYs) it provides per unit of additional cost, compared with current NHS treatment (its incremental cost-effectiveness ratio or ICER). However, ‘other factors’ are sometimes invoked in order to prioritise the needs of particular groups, even if this results in the NHS delivering fewer QALYs overall.^1 2^ One group of technologies that is systematically prioritised in this way is ultra-orphan drugs for very rare diseases^1^, appraised through NICE’s highly specialised technologies (HST) programme.^3^

Although NICE is open about the role that factors aside from cost-effectiveness play in its approach, it maintains that ‘the primary consideration underpinning our guidance and standards is the overall population need’.^2^ It also states that it ‘cannot apply the ‘rule of rescue’, which refers to the desire to help an identifiable person whose life is in danger no matter how much it costs’.^2^ This paper explores the latter claim by identifying key characteristics of the rule and establishing to what extent these are also features of NICE’s approach to appraising HSTs. It argues that although NICE in all likelihood does not act because of the rule of rescue in prioritising HSTs, these actions are nevertheless in accordance with the rule and are not explained by the full articulation of any alternative set of rationales. The implications of this finding for the transparency and fairness of NICE decision-making are briefly discussed.

## The rule of rescue

The term ‘rule of rescue’^2^ was coined by the medical ethicist Jonsen in 1986 to describe the moral challenge that life-saving technologies pose to the utilitarian ethics of health technology assessment (HTA). In Jonsen’s words:

‘Our moral response to the imminence of death demands that we rescue the doomed. We throw a rope to the drowning, rush into burning buildings to snatch the entrapped, dispatch teams to search for the snowbound. This rescue morality spills over into medical care, where our ropes are our artificial hearts, our rush is the mobile critical care units, our teams the transplant services. The imperative to rescue is, undoubtedly, of great moral significance: but the imperative seems to grow into a compulsion, more instinctive than rational.’^4^

Jonsen described the rule as a ‘deontological imperative’ and, though aware of its problematic implications, he considered it to play an inevitable role in healthcare priority setting, inferring that ‘even the most evangelical utilitarian would find it difficult to expunge the rule of rescue from the psychological dynamics’ of HTA.^4^ This was confirmed during the infamous Oregon priority-setting exercise, in which a ranking of healthcare services based on cost-effectiveness alone had to be substantially re-ordered to account for both policy-makers’ and the public’s desire to prioritise potentially life-saving treatments (such as appendectomies) over more efficient interventions (such as tooth capping).^5–7^

**Figure 1:**
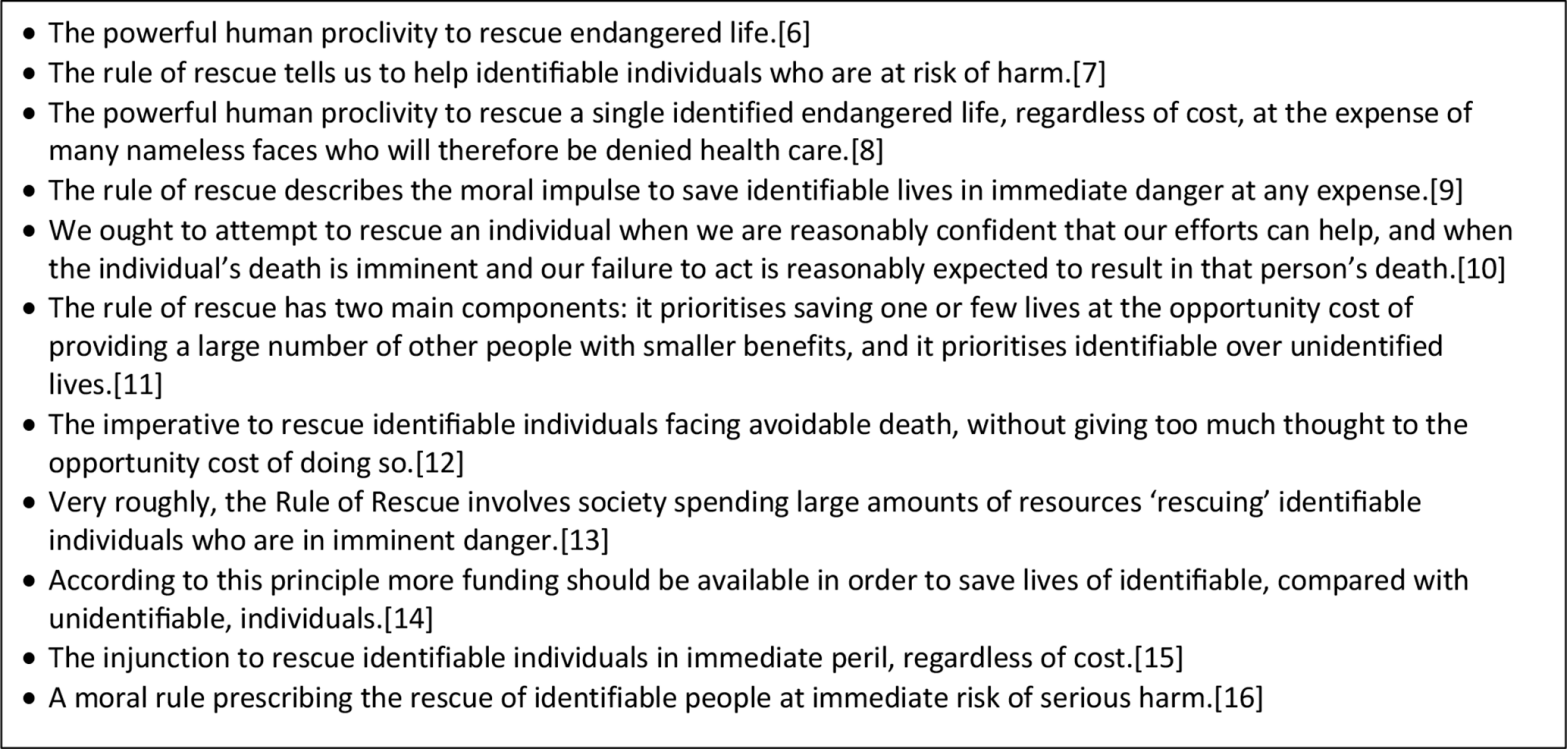
Selected definitions of the rule of rescue.^6–16^

The rule of rescue has since been interpreted in various ways, ranging from highly demanding definitions that narrowly limit its application^8–11^ to less restrictive characterisations that allow for wider adoption (figure 1).^6 7 12–15^ Definitions are generally consistent, however, in including three criteria that together might be considered to reflect the substance of Jonsen’s rule, which are:

- The intervention must offer significant benefits to those at risk of death or other serious harm.
- The beneficiaries of the rescue must be identifiable.
- The rescuer must be willing to bear significant opportunity cost in order to carry out the rescue.^3^

While some definitions describe the rule of rescue as being psychologically motivated,^6 8^ others give it the status of a normative principle.^10 14 16^ It is beyond this paper’s scope to consider whether the rule can be morally justified, but it is notable that much of the literature is sceptical on this point.^9–11 14–18^ The principle of prioritising those in greatest need, even when this leads to significant opportunity cost, is supported by strong moral arguments^19^ and is commonly adopted in HTA.^20–22^ However, the rule’s additional reliance on identifiability appears problematic. Indeed, ‘attempts at providing a morally signiﬁcant deﬁnition of identiﬁability have met formidable criticism from philosophers’, with identifiability ‘simply collaps(ing) into *salience of the victim’s need*’, which ‘does not constitute any moral reason for favouring some people over others’.^11^ Thus, while there is clearly a psychological difference between ‘Terry Q., lying injured in the passenger seat of the wrecked automobile on the corner of main street and broadway’, and ‘the person who, extrapolating from traffic records, will be in a similar, serious car accident tomorrow’,^23^ it is not clear that this often observed preference—the so-called identifiable victim effect^24^—can be morally justified.

NICE itself refers to the rule of rescue as a ‘desire’ and its interpretation of the rule is unusually narrow in being limited to scenarios in which a single identifiable person whose life is at risk is helped, with no regard given to cost.^2^ Given that NICE is statutorily required to ‘have regard to the broad balance between the benefits and costs’ of the technologies that it considers^25^ and given that its recommendations necessarily relate to populations rather than individuals, it is inevitable that NICE will not be found to adopt the rule as thus defined. However, the rule is generally understood more broadly and was coined by Jonsen to describe precisely the types of dilemma that NICE regularly confronts, perhaps most vividly in its consideration of HSTs. This paper therefore does not take NICE’s claim to reject the rule at face value, but rather explores the extent to which the HST programme might be considered to adopt the rule as it is commonly understood.

## NICE’s appraisal of HSTs

NICE’s HST programme, specifically for the appraisal of ultra-orphan drugs, was established in 2013. Previously the responsibility of the Advisory Group for National Specialised Services (AGNSS), evaluation of these technologies had historically taken account of a range of factors aside from cost-effectiveness, in recognition of the difficulties associated with developing treatments for very small patient populations.^26^ In taking over AGNSS’s role, NICE agreed that an approach ‘in which the greatest gain for the greatest number is valued highly’ would not reflect ‘the particular circumstances of these very rare conditions’.^3 27^ As such, it judged that while orphan drugs considered via the main technology appraisal programme should continue to be evaluated ‘in the same way as any other treatment’,^28^ ultra-orphan drugs considered via the HST programme would be evaluated according to different criteria.^3 27^

**Figure 2:**
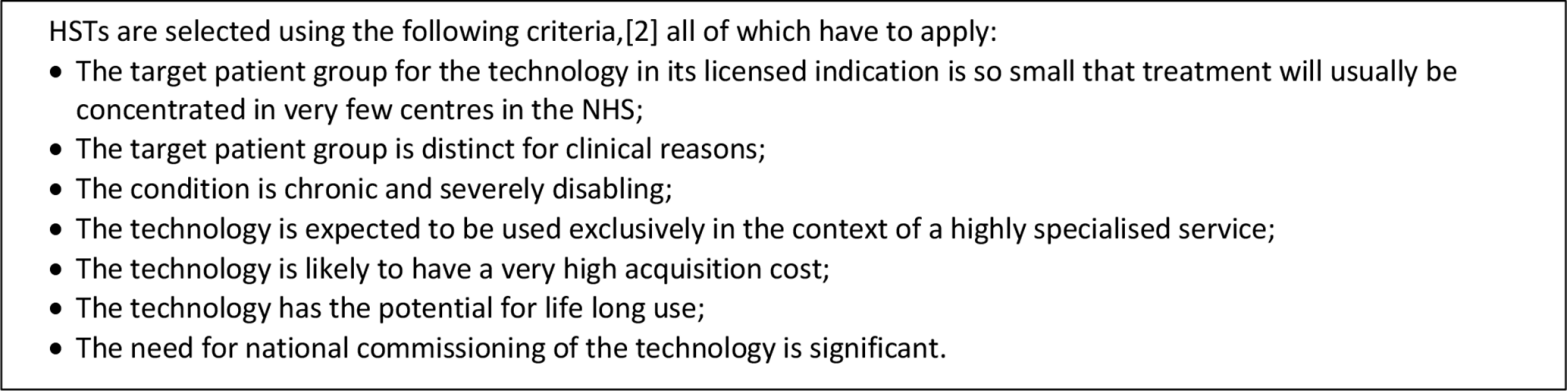
HST eligibility criteria.^2^ HST, highly specialised technology; NHS, National Health Service.

The most significant difference relates to the ICER at which a technology is deemed to be an acceptable use of NHS resources. In the main programme, the generally applied cost-effectiveness ‘threshold’ is £20 000–£30 000/QALY, increasing to £50 000/QALY for technologies that are life-extending at the end of life.^1^ In the HST programme, the equivalent figure is £100 000/QALY, increasing to £300 000/QALY for technologies that offer particularly large health gains.^3^ Given the advantage this offers to those who stand to benefit from a technology’s recommendation, appraisal via the programme is desirable for manufacturers and patient groups. However, restrictive eligibility criteria mean that only 13 drugs have been appraised as HSTs to date (figure 2). In each case, NICE recommended that the drug be adopted by the NHS.

## The HST programme and the rule of rescue

This section examines whether the HST programme might be considered to apply the rule of rescue by exploring the extent to which it fulfils the three criteria that are together taken to constitute the substance of the rule.

### Criterion 1: the intervention must offer significant benefits to those at risk of death or other serious harm

In the context of HTA, a ‘rescue’ involves providing someone who suffers from a serious medical condition with access to a potentially beneficial treatment. Understood as such, almost any effective treatment for a severe condition might be considered a form of rescue. However, most definitions of the rule demand more: the condition should be either life-threatening or severely debilitating and the intervention should be capable of either saving the endangered life or offering very considerable benefit.

Eligibility for appraisal via the HST programme is limited to conditions that are, at a minimum, ‘severely disabling’.^3^ Although this does not require that patients be at imminent risk of death, in 10 of the 13 completed HST appraisals the treated condition is associated with significantly reduced life expectancy (table 1). In each case, the treatment has been shown to potentially offer large benefits, in terms of either length of life, quality of life or both; of the 13 appraised drugs, 10 were considered likely to offer five or more additional QALYs over the course of a patient’s lifetime compared with existing treatment, and in eight cases the feasible range reached 12 QALYs or more. In comparison, research has indicated that new medicines subject to HTA typically offer a mean lifetime gain of around 0.5 QALYs.^4^ ^29^

**Table 1:** HST appraisals published to date

Reference	Technology (brand)/indication	Prognosis without treatment	Patient population size (England)	QALY gain	ICER* (£/QALY)	Recommendation†‡
HST 1^45–47^	Eculizumab (Soliris)/atypical haemolytic uraemic syndrome	Acute mortality ranges from 10% to 15%; up to 70% of patients progress to end–stage renal failure and need dialysis or die within the first year of disease.	Estimated 170 eligible patients at the time of appraisal; 20 patients per year thereafter.	10–25	Not applicable (n/a)	Unable to prepare a recommendation; recommended; **recommended**
HST 2^48–50^	Elosulfase alfa (Vimizim)/mucopolysaccharidosis type IVa	Variable. Severe disease is early onset (<3 years), with high morbidity and rapid progression. The condition leads to reduced life expectancy, primarily through respiratory failure and heart problems.	Estimated 74–77 eligible patients at the time of appraisal; three patients per year thereafter.	5–20	n/a	Minded not to recommend; recommended; **recommended (MAS)**
HST 3^51 52^	Ataluren (Translarna)/Duchenne muscular dystrophy (DMD) with a non-sense mutation in the dystrophin gene	People with DMD have a gradual decline in physical functioning, with subsequent respiratory and cardiac failure that leads to death, usually before age 30 years.	Estimated 80 eligible patients at the time of appraisal.	2.9–8.6	n/a	Minded not to recommend; **recommended (MAS, PAS)**
HST 4^53 54^	Migalastat (Galafold)/Fabry disease	Variable. The disease leads to irreversible organ damage, resulting in progressive kidney and heart disease and increased risk of stroke at a relatively young age. Fabry disease is associated with both reduced quality of life and reduced life expectancy.	Estimated 142 eligible patients at the time of appraisal.	0.3–1.0	n/a	Recommended; **recommended (PAS)**
HST 5^55 56^	Eliglustat (Cerdelga)/type 1 Gaucher disease	Variable. Gaucher disease causes symptoms such as fatigue, bone pain and reduced mobility. People who present early have a particularly poor prognosis and usually develop bone disease and immobility in the third or fourth decade of life, with a high early mortality.	Approximately 50–100 eligible patients at the time of appraisal.	1.0–1.1	n/a	Not recommended; **recommended (PAS)**
HST 6^57–59^	Asfotase alfa (Strensiq)/paediatric-onset hypophosphatasia	Symptoms are variable but include chronic debilitating pain, rickets, softening and weakening of the bones, bone deformity and a greater incidence of fractures. The most severe forms tend to occur before birth and in early infancy. About 50%–100% of babies presenting with the condition die within the first year of life.	Between 1 and 7 eligible patients per year.	14–25	n/a	Not recommended; recommended (perinatal-onset and infantile-onset only); **recommended (all paediatric onset, PAS, MAS)**
HST 7^60 61^	Strimvelis/adenosine deaminase deficiency–severe combined immunodeficiency	The main feature of ADA–SCID is a severely compromised immune system, which, if untreated, requires the patient to be isolated, severely impacting quality of life. Untreated infants typically die before school age.	The company estimated that three people a year are diagnosed with ADA–SCID and that one person a year would have Strimvelis treatment.	14.0–19.6	<1 20 506	Recommended; **recommended**
HST 8^62 63^	Burosumab (Crysvita)/X-linked hypophosphataemia in children and young people	Early signs include skeletal abnormalities such as bowed or bent legs, below average height and irregular growth of the skull. Bone defects are common in children and can cause pain and subsequently limit physical functioning. When bone growth stops, bone deformities become irreversible and can be the source of continuing pain.	Up to 250 eligible UK patients at the time of appraisal.	5.5–16.0	1 13 000–1 50 000	Not recommended; **recommended (PAS)**
HST 9^64 65^	Inotersen (Tegsedi)/hereditary transthyretin amyloidosis (hATTR)	People may have a range of symptoms affecting one or more body systems. These can include the autonomic nervous system, peripheral nerves, heart, gastrointestinal system, eyes and central nervous system. The effects and complications of the condition can lead to death within 3–15 years of symptoms developing.	Approximately 150 eligible UK patients at the time of appraisal.	<10	96 697	Not recommended; **recommended (PAS)**
HST 10^66 67^	Patisiran (Onpattro)/hATTR	As above.	As above.	9.2–12.2	80 730–1 25 256	Not recommended; **recommended (PAS)**
HST 11^68^	Voretigene neparvovec (Luxturna)/inherited retinal dystrophies caused by RPE65 gene mutations	People with the condition have progressive vision loss, beginning as early as the first few months of life, or during childhood, or adolescence. Ultimately, the deterioration leads to near-total blindness.	Approximately 86 eligible patients at the time of appraisal.	12.1–17.7	1 14 956–1 55 750 (pre discount)§	**Recommended (PAS)**
HST 12^69–71^	Cerliponase alfa (Brineura)/neuronal ceroid lipofuscinosis type 2	Symptoms include a decline in mental and other capacities, epilepsy and sight loss in late infancy, leading to death by early adolescence. Symptoms appear in the second year of life and can then progress rapidly. Ultimately, the child will become totally dependent on family and carers for all their needs.	Approximately 30–50 eligible UK patients at the time of appraisal; 3–6 patients per year thereafter.	>30	>3 00 000 (pre discount)§	Not recommended; not recommended; **recommended (MAS)**
HST 13^72 73^	Volanesorsen (Waylivra)/familial chylomicronaemia syndrome	Symptoms include repeated episodes of severe abdominal pain, recurrent episodes of acute pancreatitis, liver and spleen enlargement, and fatigue. Acute pancreatitis is a life-threatening condition for which intensive care may be needed.	Approximately 80–100 eligible UK patients at the time of appraisal.	<10	98 103	Not recommended; **recommended (PAS)**

*Calculation of a technology’s incremental cost-effectiveness ratio (ICER) did not form part of the formal methods of the HST process at the time that HTS1–HST6 took place.

†Provisional decisions in normal type, final decisions indicated by bold type.

‡A PAS is the standard way for pharmaceutical companies to make high-cost drugs affordable for the NHS when they are routinely commissioned. Each scheme is approved by the Department of Health. They can be a simple discount or more complex (eg, price cap). A managed access scheme consists of two components: (1) a data collection arrangement, which sets out data that will be collected to resolve clinical uncertainty during the 'managed access' period, and (2) a commercial agreement that determines how much the NHS will pay for the treatment during the managed access period. This could be a PAS.

§ICERs for these appraisals are commercial in confidence and the figures quoted do not reflect the price paid by the NHS after the agreed discount has been applied.

ADA–SCID, adenosine deaminase deficiency–severe combined immunodeficiency; HST, highly specialised technology; MAS, managed access scheme; NHS, National Health Service; PAS, patient access scheme; QALY, quality-adjusted life-year.

This potential for large health gains is in part a product of the HST programme’s focus on severely disabling conditions, since patients suffering from these conditions are likely to be in a poor state of health, providing a low QALY baseline against which the benefits of treatment will be compared. In addition, the high cost of many ultra-orphan drugs makes it unlikely that such products would be brought to market unless there was a reasonable prospect of their prices being justified by significant clinical benefits. Thus, the design and scope of the HST programme make it extremely likely that the technologies it evaluates will offer significant benefits to those at risk of death or other serious harm—an expectation that has so far been borne out in practice.

### Criterion 2: the beneficiaries of the rescue must be identifiable

As a national priority setter, NICE’s advice invariably relates to patient populations rather than individuals. However, the beneficiaries of its recommendations are arguably always identifiable because their eligibility for treatment with a given technology distinguishes them from other NHS users. In contrast, those NHS users who bear the opportunity cost of NICE’s recommendations are entirely unidentifiable, because NICE has no knowledge of what will be displaced in order to fund its recommendations. As such, it could be argued that all NICE appraisals meet this criterion, regardless of the size of the affected population.

Alternatively, it could be argued that NICE’s appraisals never meet this criterion, because its recommendations apply to both identifiable current patients and unidentifiable future patients who have not yet been diagnosed (or perhaps even born). However, the purpose here is not to suggest that NICE’s appraisals meet all possible standards of identifiability, but is only that they meet the relatively weak standard implied by the rule of rescue. In making this argument, a comparison can be drawn with Jonsen’s formative example of the artificial heart, which ‘might annually bring 4 years of extended life to some 25 000 persons’ with cardiomyopathy at the expense of the ‘invisible multitudes’ who die of other conditions, and a typical NICE recommendation, which benefits a few thousand patients while exacting an opportunity cost on millions of unknown NHS users.^4^ Considered in this light, even though NICE’s recommendations apply to both current and future patients, it is difficult to argue that they fail to meet the standard of identifiability intended by Jonsen when he coined the rule of rescue.

The potential beneficiaries of ultra-orphan drugs are also identifiable in a stronger sense much closer to that of ‘Terry Q.’, lying injured at the side of the road, in need of rescue.^23^ Across the 13 HST appraisals completed to date, the most common condition affected an estimated 250 UK patients at the time of appraisal, with several recommendations applying to fewer than 10 new patients annually (table 1). In one case, it was anticipated that the treatment would be given to a single patient each year.^5^ Such small group sizes, combined with the distinctive and serious nature of the related conditions, make these patients highly identifiable and their needs especially salient. This salience is further enhanced by NICE’s practice of inviting patients and carers to describe their experiences through oral and written testimony. While such engagement is not unique to HSTs, the exceptionally small patient populations involved in these appraisals mean that decision-makers have direct contact with a much larger proportion of those who stand to benefit, reducing group anonymity and further increasing the extent to which the beneficiaries might come to feel ‘known’. Thus, while all NICE’s appraisals could be considered to meet the standard of identifiability required by Jonsen, those whose needs are addressed via the HST programme are also identifiable in sense much more likely to elicit the identifiable victim effect.^24 30^

### Criterion 3: the rescuer must be willing to bear significant opportunity cost in order to carry out the rescue

According to NICE’s current methods, HSTs will generally be recommended up to an ICER of £100 000/QALY, increasing to £300 000/QALY if the expected health gain is sufficiently large.^3^ In comparison, NICE’s standard cost-effectiveness threshold is £20 000–£30 000/QALY,^1^ with empirical evidence suggesting that the actual cost to the NHS of producing a single QALY lies between £5000 and £15 000.^31–33^ As such, for every QALY gained at a cost of £100 000–£300 000, between 6 and 60 QALYs are likely displaced from the NHS as a whole. Thus, while it cannot be claimed that the HST programme pays no regard to cost—(to do so would contravene NICE’s statutory obligations)—its methods allow NICE to commit the NHS to very significant opportunity costs in order to provide access to these drugs.

**Figure 3:**
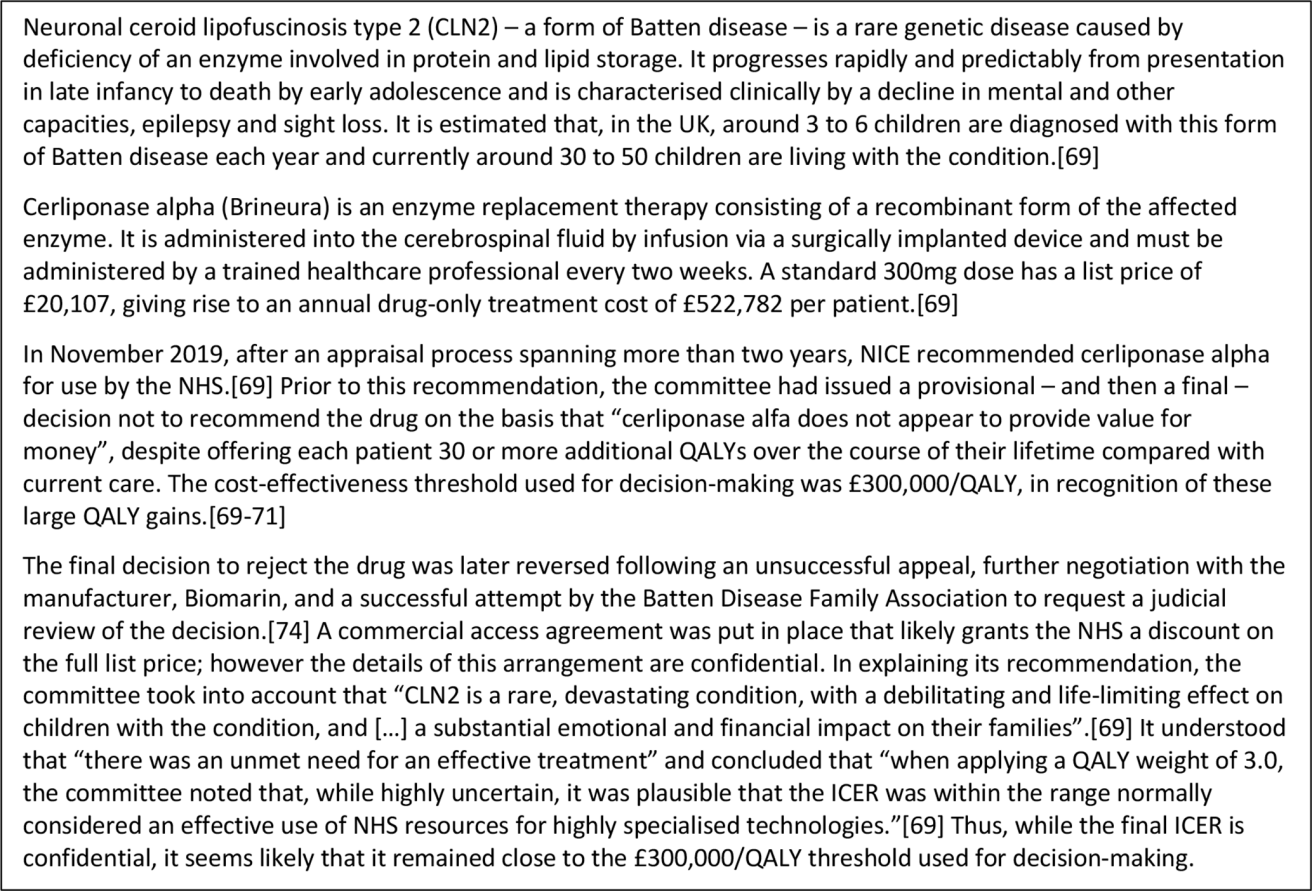
Cerliponase alpha for the treatment of Batten disease^69–71 74^. ICER, incremental cost-effectiveness ratio; NHS, National Health Service; NICE, National Institute for Health and Care Excellence; QALY, quality-adjusted life-year.

Decisions made by the programme to date confirm NICE’s willingness to accept this magnitude of opportunity cost in practice as well as policy. Although exact ICERs are often commercial in confidence due to price discounts secured following negotiation, several drugs have been recommended at a cost of around £100 000/QALY, and, in one recent case, the final ICER appears to have been in the region of £300 000/QALY (table 1 and figure 3). Indeed, despite evaluating some of the most expensive drugs ever to have been brought to market and despite in several cases provisionally judging them to offer insufficient value for money to warrant recommendation, NICE has never rejected an HST. Thus, while there may be a point at which NICE is willing to turn its back on the potential ‘rescues’ offered by such drugs, that point has evidently not yet been reached in practice.

## Discussion

The rule of rescue is an amorphous concept that can be defined and used in a variety of ways. In claiming to reject the rule, NICE adopts a particularly narrow interpretation that is, in practical terms, incompatible with its role as a national priority setter. However, this rejection is in tension with an approach to appraising HSTs that might reasonably be considered to reflect the substance of the rule, as it is commonly understood. As such, while NICE’s claim that it ‘cannot apply’ the rule of rescue should not be considered wrong, it also cannot be considered entirely right.^2^

That is not to suggest that in its design and conduct of the HST programme, NICE has sought to adopt the rule of rescue. That is, in Kantian terms, it is not argued that in facilitating access to these technologies NICE acts for the sake of the rule.^34^ Rather, it is suggested that these actions are in accordance with the rule, while perhaps being motivated by a more complex set of normative considerations. This is an important distinction because the moral basis for the rule of rescue itself appears, at best, questionable.^9–11 14–18^ In contrast, it is perfectly possible that the reasons that lead NICE to act in accordance with the rule provide a morally sound basis for decision-making.

NICE, however, is not explicit about these reasons. In describing its approach to HSTs, NICE refers to the ‘particular circumstances of these very rare conditions’, specifically ‘the vulnerability of very small patient groups with limited treatment options, the nature and extent of the evidence and the challenge for manufacturers in making a reasonable return on their research and development investment’.^3 27^ The programme’s focus on conditions that are ‘chronic and severely disabling’ suggests that weight is also given to the severity of clinical need.^3^ But while NICE draws attention to these considerations, it is not explicit about the role they play in its approach to HSTs. It is therefore left to us to speculate as to why NICE considers these technologies worthy of such significant prioritisation compared with the many other technologies that it appraises.

A clue is offered by the other circumstances in which NICE is willing to exceed its usual £20 000–£30 000/QALY cost-effectiveness threshold. The so-called ‘end-of-life rules’, introduced in 2009, allow appraisal committees to ‘give greater weight to QALYs achieved in the later stages of terminal diseases’, effectively raising the threshold for some life-extending technologies to £50 000/QALY.^1 35^ The needs of severely ill young patients with limited treatment options are prioritised through the lower discount rate applied in cases in which large health gains are ‘sustained over a very long period (normally at least 30 years)’, and appraisal committees are also encouraged to give special consideration to the ‘innovative nature’ of technologies that may not otherwise be deemed cost-effective.^1^ Each of these prioritisation mechanisms are potentially applicable to HSTs, when the relevant criteria are met. There is also precedent for facilitating access to drugs for which ‘the uncertainty in the clinical and cost-effectiveness data is too great to recommend the drug for routine use’, through the special arrangements in place for NICE’s operation of the Cancer Drugs Fund.^35^ Indeed, drugs approved through the HST programme are often subject to similar managed access arrangements in which further data collection is a condition of the NHS’s adoption of a technology (table 1). None of these mechanisms, however, grant technologies sufficient priority to facilitate their recommendation at the >£100 000/QALY ICERs typical of the HST programme. NICE’s exceptionalisation of HSTs must therefore be based—at least in part—on other reasons.

The obvious candidate is rarity. Various bodies, including the UK Medicines and Healthcare products Regulatory Agency, confer special treatment on orphan and ultra-orphan drugs ‘to encourage the development of medicines in rare diseases’.^36^ NICE’s concern with ‘the challenge for companies in making a reasonable return’ on their investment suggests that its prioritisation of HSTs might be motivated by a similar desire to drive drug development.^3^ However, NICE does not make it clear why it considers either the development or adoption of ultra-orphan drugs to be a particular priority. This is not to say that reasonable justifications for such a view do not exist. A proponent of a rights-based sufficientarian approach,^37^ for example, might assert that all individuals in a society are entitled to a decent minimum level of health (or healthcare) and that rare disease patients falling below this level should therefore be provided with access to treatment, whatever the cost. But NICE does not adopt a rights-based approach and does not otherwise take account of notions of sufficiency in its decision-making. Alternatively, a luck egalitarian^38^ might point out the misfortune of requiring treatment with an extremely expensive ultra-orphan drug and argue that resources should allocated in order to eliminate the disadvantage derived from such ‘brute luck’. However, this seems incompatible with NICE’s broadly utilitarian approach, which though sympathetic to the desirability of reducing health inequalities, generally considers allocative efficiency to be of greater importance. A promising argument for specifically prioritising conditions that are both rare and severe is offered by the notion of aggregate relevant claims, which suggests that any level of opportunity cost might be considered acceptable in helping the very sick, as long as the health burdens of those who suffer the cost are sufficiently small in comparison.^6^ ^39^ However, NICE’s approach is generally insensitive to (and, in fact, ignorant of) the nature of the health displaced by its recommendations. Indeed, none of these potential rationales are compatible with NICE’s general approach or able to explain why, under this approach, ultra-orphan drugs are granted substantial priority, while orphan drugs are granted none.

In the absence of a clear normative reason for NICE’s exceptionalisation of HSTs, consideration must be given to other possible explanations. The cases considered through the HST programme tend to be highly emotive and distressing, often featuring young, vulnerable patients who suffer the double misfortune of having a life-limiting illness that is both very severe and very rare. In inheriting responsibility for the evaluation of ultra-orphan drugs from AGNSS, NICE was faced with the choice of either continuing to prioritise these technologies or publicly justifying why it does not consider them to offer sufficient value for money. It is easy to understand why it might have been considered politically expedient to choose the former option, even if this did not easily accord with NICE’s wider approach. An argument could also be made that, while NICE decision-makers do not prioritise HSTs because of the rule of rescue and do not accept the rule as a normative principle, both are driven by the same psychological desire to ‘rescue the doomed’.^4^ That is, in enacting a policy that—in an affordably small number of cases—substantially prioritises the needs of a highly identifiable and unfortunate few over the less salient many, NICE has itself succumbed to the identifiable victim effect.

Whatever its reasons for acting as it does, the disparity between NICE’s explicit rejection of the rule of rescue and its apparent accordance with the rule in its treatment of HSTs potentially undermines the fairness of NICE’s approach. This has historically been based largely on the notion of procedural justice and NICE’s commitment to observing the terms of Daniels and Sabin’s accountability for reasonableness framework, which requires—among other things—that both priority-setting decisions and their rationales be made public.^28 40^ However, as has been illustrated, NICE does not make public its reasons for prioritising HSTs. It could also be argued that in specifically reaffirming its rejection of the rule of rescue in its new statement of principles, while failing in the same document to explicitly acknowledge or explain the basis for its prioritisation of HSTs, NICE adopts a strategy that purposefully obscures a problematic aspect of its approach. That is, it could be suggested that in claiming to reject the rule of rescue, NICE implies that it is able to respond to the moral complexities of these ‘rescue’ scenarios with its basic approach intact, when, in fact, the HST programme provides it with a means of ‘muddling through’^41–43^ these challenging cases only by substantially departing from this approach.

While this pragmatic strategy is understandable given the difficult job that NICE is tasked with, the resultant loss of transparency undermines its claim to act as a fair decision-maker, putting the legitimacy of its decisions at risk. It also relieves NICE of the immediate need to resolve apparent inconsistencies in its approach and to publicly account for the normative basis of some of its most contentious decisions. If NICE stands by these decisions, it must be willing to explain the true basis on which they have been made. In doing so, it is likely to find that reference to the rule of rescue, already unhelpful, becomes unnecessary.

## Data Availability

All data relevant to the study are included in the article.

## Footnotes

Orphan drugs are usually defined as drugs in which the prevalence of the condition for which they are indicated does not exceed 5 in 10 000 patients (ie, 1 in 2000). Ultra-orphan drugs are typically defined as drugs for which prevalence does not exceed 1 in 50 000 patients.^44^

A distinction is drawn in this paper between the ‘rule of rescue’ and the ‘duty of easy rescue’, which limits the moral relevance of any duty to rescue to cases in which a person can prevent serious harm to another at minimal cost to themselves.^11^ Given that NICE specifically describes its position in relation to the former rather than the latter, this paper does the same.

These same criteria were identified by Largent and Pearson, who state: ‘There are at least three constituent parts of the rule of rescue—identifiable individuals, endangered lives and opportunity costs’.^9^

Based on new medicines appraised through the Scottish Medicine Consortium—which has a similar scope to the NICE core appraisal programme—between 2001 and 2010.

As part of NICE’s 2017/18 appraisal of Strimvelis for treating adenosine deaminase deficiency–severe combined immunodeficiency (ADA–SCID, also known as ‘bubble baby’ disease because of the extreme vulnerability of its sufferers to infection), the company estimated that ‘three people a year would be diagnosed with ADA–SCID in England’. Of these, it was expected that one person would be treated with Strimvelis at a cost of approximately £500 000.

For example, if the health displaced in order to fund an HST relates to services to relieve mild pain caused by ingrown toenails – a relatively small burden that might be considered normatively irrelevant in comparison with the burden suffered by the HST patient.
